# Treatment in Septic Conditions

**Published:** 1910-03-19

**Authors:** Edred M. Corner

**Affiliations:** Surgeon in Charge of Out-Patients, St. Thomas' Hospital; Surgeon, Hospital for Sick Children


### Hospital Clinics.

### TREATMENT IN SEPTIC CONDITIONS.

By EDRED M. CORNER, B.Sc., M.B., M.C., F.R.C.S.; Surgeon in Charge of Out-Patients, St. Thomas' Hospital; Surgeon, Hospital for Sick Children.

(A Lecture delivered at the Polyclinic, Chenies Street, on January 25.)

This evening I should like to mention a few points in connection with the treatment of the common septic conditions. The reason why I have chosen this subject is because during the last three years or so I have had under my inspection about thirty-five of the beds at St. Thomas's Hospital which are devoted to patients suffering from these septic diseases. In that space of time a large amount of material has passed under my supervision, and I thought the time ripe to gather together some observations upon these cases.

Curiously enough the first point that I would make to-day is one that I ran across last; indeed, I did not realise its importance in the superlative degree until a few weeks ago. This point is the necessity of *rest*. We are apt to assume that rest is always made the backbone of the treatment in acute conditions, but recently I had the point thrust upon my notice that rest is not always enjoined as it should be. One of these cases, for instance, was that of a young woman who suffered from an ordinary septic leg. It was the kind of condition that would readily yield to rest and hot dressings. But she had been treated only with hot dressings, and had very little rest. After about a week or ten days it became evident that she was not getting on at all well and she was admitted to hospital. Here the hot dressings were continued in addition to rest in bed, and she got quite well. The first point, therefore, is the necessity for rest in these acute cases.

### Fomentations and Incisions.

The next point is that of *hot dressings*. There is no doubt that hot dressings, which are the modern and cleanly descendant of the old-fashioned poultice, do a great amount of good, chiefly by stimulating the circulation, not only in the part where the hot dressing is applied, but in other parts as well; and not only the circulation of the blood, but also that of the lymph. Probably they also stimulate the activity of the cells in the region involved. Therefore the backbone of the treatment of septic diseases is, first, rest, and, secondly, hot dressings. A large proportion of the cases get quite well under this simple treatment.

I can now proceed to the consideration of some further points which I have noticed in dealing with the cases brought to these septic beds. The percentage of the hospital cases in which an *incision* is necessary, in order to assist the hot dressings and the rest, is necessarily very high, and for this reason: that if the cases get well with ordinary care, such as rest and hot dressings, the patients are, as a rule, not admitted to the hospital. These incisions have a number of uses. The most important use is in the letting out of material which, one may say, clogs the processes of repair. That material, such as pus, removed, the part is better able to fight its own battle. But from time to time—indeed, very frequently when the patients are admitted to the hospital—it is necessary to do something more than these simple medical and surgical measures, and it is to these additions that I would particularly commend your attention.

### The Hyperæmic Method.

One of these I have already mentioned in a lecture in this room—namely, the method of treating septic wounds and septic foci by means of passive congestion. The uses of passive congestion, like most other things, when it was first introduced were grossly exaggerated. It was even suggested that its advent would supersede the surgical knife. That, of course, is quite untrue. Now that passive congestion has been in use for some time, and its results can be submitted to calm criticism, it is found that the method of passive congestion has a number of failures. One of its great failures is in erysipelas, in which condition it does no good at all. The inflammation will travel as high as, or will even pass, the bandage which has been used to produce the passive congestion. And again, in a certain number of cases of septic infection of the joints and in gonorrhœal arthritis, passive congestion increases the amount of pain and does more harm than good. Nevertheless, passive congestion is a form of treatment very easily utilised, and it does possess advantages.

Let us consider it first as applied to the limbs. It is produced by applying a bandage around the upper part of the arm or the leg. That elastic bandage must not be tight enough in any way to interfere with the arterial pulse below it, and must only be sufficient to impede, not to stop, the return of the blood in the veins. It also impedes the lymphatics. As a result, the limb below the bandage becomes somewhat swollen. The difficulty in applying the treatment is the impossibility of finding any trustworthy guide as to how tight this bandage should be applied in a particular case. Indeed, the degree of tightness varies with the case. In some people it has to be applied more tightly than in others. It may require two or three, or even half a dozen applications of the bandage before the proper degree of tightness is obtained, and it is often found that the patient's statement as to the tightness of the bandage is a more trustworthy guide than the random attempts of the practitioner.

From the mention of this difficulty I pass to the consideration of the great advantage which this form of treatment possesses. The advantage lies in the fact that, as soon as the right tightness is obtained, the medical man will know within an hour whether or not his treatment is going to do good. The patient will lose his bodily pain within, perhaps, ten minutes of the application of the bandage. Previous to its application the patient has been worn out with pain and sleeplessness, and as a consequence has no appetite. If that pain can be relieved—and it may often be relieved within ten minutes by this method—the patient will immediately drop to sleep—not the heavy, restless sleep such as one sees in feverish patients with high temperatures, but a fresh, wholesome sleep; and on waking in perhaps two hours' time he will feel much happier and perhaps take a good meal.

A further advantage is brought about by the impeding of the venous and lymphatic return by means of the bandage, while the arterial pulse is not affected. In the venous return the poisonous material is carried from the site of the disease to other parts of the body, and therefore, with the application of the bandage, the patient is getting a far smaller dose of the poison than before. Consequently within one or two hours the patient's temperature will come down from, perhaps, 103° to 99°. In a similar manner the pulse-rate will also come down.

The treatment is easy of application; indeed, the patients can frequently carry it out for themselves; and altogether it is well worth trying.

### Treatment by Suction.

Since people found that in certain cases—not in all—great benefit is derived from passive congestion as applied to the limb, it has been the object of many to apply passive congestion to septic foci on the trunk or neck or head. It is quite obvious that bands cannot be applied to the trunk. Passive congestion has, however, been produced in the scalp by means of a bandage round the head, and in the face by a bandage round the neck. These are not methods which recommend themselves to patients, and, moreover, from the clinical point of view I have never seen such good results follow the application of the bandage round the head or neck as round the arm or the thigh. Therefore we have fallen back upon other methods.

Passive congestion is applied to the trunk by means of cups, really a revivification of the old method of "cupping," but with modern appliances. A cup is used somewhat like an inverted wineglass with a hollow stem. Attached to that hollow stem is a rubber bulb which when squeezed will expel the air, and the patient's skin having been anointed with vaseline or something similar a vaccum is created inside the cup. By this means we can apply suction and a certain amount of passive congestion to the trunk, but it is obvious that we can only do it for small foci. But the cure of some septic foci on the trunk can be very greatly expedited by this method. Not only does the passive congestion itself help to relieve the trouble, but the suction also gives assistance to the patient.

Up to the present I have talked of passive congestion as combined with rest and hot dressings. I can now pass on to speak of passive congestion as combined with rest, hot dressings, and an incision. It is absolutely proved that with passive congestion, if an incision is required at all, it must be a very small incision. Whereas an incision of two or three inches would have been made in former days, now, with passive congestion, the patient may get quite well with an incision of half an inch. If, for example, the incision is made in the leg, and passive congestion is applied in the upper part of the thigh, certain changes will take place of which it is as well to be aware beforehand. The granulations or the tissues in the incision will become blue, cyanosed, and œdematous. I take special care to mention that result because when people for the first time see the incision looking blue and pouting they receive an impression that the wound is worse than it is. To sum up, in medical cases which get well without incision, and in surgical cases in which incision is necessary, treatment by passive congestion can be employed, either by means of bands on the limbs or by cups on the trunk. And when the incision is made on the trunk there is the added advantage of suction.

### Vaccines.

I pass now to speak about the effect I have found from modern methods of treatment, as, for instance, *vaccines*. Vaccines in the treatment of septic conditions have been employed for some years. I have used them in a large number of cases, so that I ought to be in a position to form some estimate of their value. I may sum up the situation by saying that I am exceedingly disappointed in the results. One of the things I hoped for when I undertook the inspection of these beds was the possibility of formulating some conclusions as to the cases in which vaccines did good and those in which they did none. That hope has been quite unrealised. We have given vaccines in a large number of septic cases, and some have recovered and others have not, and it is quite impossible to say whether the recovery in the one case and the non-recovery in the other are to be attributed to the vaccines. Doubtless some partisans of vaccine therapy would say that in the cases which recover the result is due to the vaccines, and in the cases which do not recover the blame is to be laid upon the virulence of the infection. This attitude however I regard as not sportsmanlike. It ought to add a certain amount of weight to one's words when it is said that in St. Thomas's the vaccine treatment of septic cases has been distinctly disappointing. Erysipelas is a septic disease, and vaccine treatment has been tried by various methods, but it has not done the least good in the world. We tried vaccines also with carbuncles. Carbuncles are usually due to a *staphylococcus*, sometimes to a mixed infection of a *staphylococcus* and a *streptococcus*. The vaccine treatment has not affected the small mortality from carbuncles. It may be asked whether it has lessened the length of the disease and permitted an earlier recovery. If we desire to estimate the rapidity of recovery we may take as a rough index the length of time which the patient has spent in the hospital. In St. Thomas's Hospital we have cases indexed for the last 20 or 30 years, before the vaccine treatment; and with the vaccine treatment, there is no appreciable difference in the time spent in the hospital by those who had vaccines and those who had not. There has not, therefore, been any particular success with vaccines in the treatment of carbuncles. With regard to the treatment of other septic conditions, septicæmia, pyæmia, and septic bone disease, the results have been equally indifferent. At first we gave vaccines in all such cases, without any obvious improvement and afterwards we gave vaccines in only 50 per cent. of the cases. The people who did not have vaccines got well just as quickly as those who had, so that on the whole the vaccine treatment has been disappointing. Of course, in many cases vaccines were given and the abscess also opened, and in these cases when the temperature fell from 103 to 99, who was to say whether the result was due to the vaccine or to the opening of the abscess? So far as one can ascertain, vaccine treatment may be of assistance to these patients; but it is certainly not a cure in itself. I remember a young doctor who had what turned out to be a staphylococcus aureus infection of the hair follicles at the back of the neck. He was perfectly willing to be a subject of experiment, and we put in a fine trocar and got out sufficient of the pus to make a culture. We made a vaccine from the growth from that staphylococcus, and vaccinated him, continuing the experiment for a sufficient length of time to show conclusively that with vaccine alone we were not going to cure him. Surgical treatment was then resorted to, the boil incised, and the patient got quite well. That is not the only case in which the matter has been put to the proof in that experimental way, and it appears certain that if vaccine is to be tried it must be combined with surgical measures, in which case it is impossible to say clinically how much of the good result is due to the vaccine and how much to the surgical treatment.

The reply that the upholders of vaccine treatment may make to this state of affairs is that the clinical results vary, of course, with the character of the infection. We have made a practice of having a culture made from the patient's own vaccine, and in that connection we ran across one or two extraordinary things which helped to throw some distrust on the efficacy of this form of treatment. For instance, one can take the ordinary acne infection of the hair follicles of the skin—some patients who have bad infections of the skin are admitted to the hospital—and have been vaccinated with a vaccine made from their own organism, without any improvement. Afterwards one may give them a stock vaccine, made from someone else's organism; and they got quite well. As another instance, we may take gonorrhœal cases. If the patients are inoculated in turn from their own vaccine and from stock vaccine, in some cases their own vaccine will do good, and in other cases the stock vaccine. In the majority of cases neither vaccine does good. Therefore it becomes a moot point as to how the character of the infection affects the vaccine treatment and its efficacy. But this much I will say, that I have never seen any harm follow the vaccine treatment, except when it has been continued for a long time, in which cases the patients have suffered from headache, anæmia, lassitude, and so forth. I can dismiss this part of the subject with one further statement. Having never seen harm follow the vaccine treatment, I would personally almost always use it, because after all it may do good, and on that entirely negative ground its use may be advocated.

### The Bacteriological Aspect.

I turn now to the question of septicæmia when the organisms are found in the blood. As a general rule we say that such a case will end fatally; but in recent times there has been an attempt to assist that septic patient by injecting a certain amount of fluid —say saline—into the loin or thigh. We know that if this fluid is injected into a certain part we create a focus of irritation there, and in consequence an abscess is formed, in which we find the septicæmic organism. The enthusiasts who initiated this treatment called these abscesses "fixation abscesses." They said that the organism was loose in the blood, and by means of "fixation abscesses" one could "fix" the organism. We have tried this method, and have certainly obtained the septicæmic organism, and have seen the patient recover. But whether the determining factor in the recovery has been the abscess or the patient's own constitution it is difficult to say.

### Pyæmia.

Pyæmic cases are certainly some of the most interesting that one can run across. I have only time to give one or two instances. One is that of a big, heavy man of 45-50, who was admitted to the hospital with a big carbuncle in his neck. He developed signs of pyæmia, the first sign being a swelling first of one shoulder-joint and then of the other. When the first shoulder-joint was attacked we used the trocar and the aspirator, and from the extraction grew an organism, the staphylococcus aureus. The joint was incised and drained. The other shoulder-joint also swelled, and we were disinclined to make an incision again. We tapped this second shoulder-joint and once more proved the presence of the pyæmic organism from the fluid. Rather to our surprise, the man started to improve, and the shoulder-joint which we tapped and from which we grew the organism never refilled. During the convalescence the fluid appeared in the knee-joint, the pyæmic organism was again found, and the joint never filled. The man entirely recovered. I saw him a year or eighteen months after his recovery, and found that the shoulder-joint which we tapped and from which we grew the organism was a very good shoulder-joint, and the knee-joint which had been affected was as good as the other knee-joint which had shown no signs of trouble. We have absolutely proved that although the organisms are present in these pyæmic foci, not only may the patient recover, but the part also may recover its function.

It has been known for many years that in pyæmia from time to time a localised swelling or blush is seen, and if it is left alone it disappears. Every now and then we have had these occurrences, and if the house surgeon was available the swelling was incised and the culture taken, the pyæmic organism being grown, from the fluid exuded. But if the house surgeon was not available no incisions were made without, apparently, affecting the case. In pyæmia the body is full of organisms which may appear in a part and then disappear and the part get quite well. Without doubt a change will come over the treatment of pyæmic cases, and we shall incise the big foci, but confine ourselves to tapping and aspirating the small foci. There will be a number of men, particularly among those who have never been able to watch a large number of septic cases, who will say that this is a retrograde step. But I do not think it is. We now know that what may be called medical rheumatism is a disease produced by a streptococcus, and rheumatic joints can get quite well without surgical treatment. Pyæmic joints are similar in character. In both instances the infection is through the bloodstream, and with pyæmic organisms in a joint that joint need not necessarily become disorganised. I argue, therefore, that in the future there will be a great change in the treatment of pyæmia.

### Glycosuria in Septic Conditions.

My final point really opens up a big subject, but a subject which is frequently thrust upon my notice when receiving reports upon these "septic" beds. We get glycosuria in septic cases, and it is quite a common thing in a patient who is suffering from a septic illness, particularly if he is run down in his general health, to discover this condition. When that glycosuria is suddenly discovered we are confronted with the problem as to whether it is diabetic or not. In the first place, it has been proved up to the hilt that almost every disease, particularly a septic disease, and I might even say every depreciation of health, is associated with the lowering of the saturation point of tissues to sugar. We can take a healthy person, feed him with glucose up to perhaps 1,500 grammes, and get a dietetic glycosuria. But with a person of lower vitality only a dose of 1,000 grammes would be necessary to produce the same result, and a person who was ill and whose resistance was less might show it at 100. Septic disease itself may cause the glycosuria from a patient's own diet or own tissues. There is a symptomatic glycosuria associated with septic diseases which may completely clear up as the patient improves. It happens from time to time that a patient is brought into the hospital, tested, and, no sugar being found, is put on the ordinary hospital diet. In a week or ten days the house surgeon may report that the patient has a large amount of glycosuria. For instance, in a case at present in the wards the amount of sugar is now very great, and yet in this case it was reported when the patient came in that there was no glycosuria. The fact is that in the hospital the patient may get a dietetic glycosuria which will clear up before the patient leaves the wards. The occurrence of the glycosuria is a distinct indication that the man's general condition is depreciated, and probably that the septic disease from which he is suffering is worse. The indication, therefore, is for active treatment. In other cases the glycosuria is due to a definite disease called diabetes, and in these cases I would also urge that its occurrence is again an indication for active treatment, and the earlier and the more energetic the treatment undertaken in diabetic cases the better for the patient. It is not worth the doctor's or the patient's while to expend a large amount of time to save a foot, for example, which is affected. It is a great deal better from the point of view of the patient's general health that an amputation should be done. In such a case the sugar in the urine, which is from the diabetes, becomes markedly less in amount and the patient's general health is very greatly improved. The result is far better than if the patient underwent a long illness in the hope of staving off amputation. As a general rule, therefore, the discovery of sugar in the urine is an indication for more energetic treatment.

